# The weakening effect of soluble epoxide hydrolase inhibitor AUDA on febrile response to lipopolysaccharide and turpentine in rat

**DOI:** 10.1007/s13105-017-0584-y

**Published:** 2017-07-24

**Authors:** Jakub Piotrowski, Tomasz Jędrzejewski, Małgorzata Pawlikowska, Agata Joanna Pacuła, Jacek Ścianowski, Wiesław Kozak

**Affiliations:** 1 Department of Immunology, Faculty of Biology and Environment Protection, Nicolaus Copernicus University, 1 Lwowska Street, 87-100 Torun, Poland; 2 Department of Organic Chemistry, Faculty of Chemistry, Nicolaus Copernicus University, 7 Gagarina Street, 87-100 Torun, Poland

**Keywords:** Inflammation, Fever, EETs, Anti-pyretic, Soluble epoxide hydrolase, Biotelemetry

## Abstract

A still growing body of evidence suggests the importance of epoxyeicosatrienoic acids (EETs) in the regulation of inflammatory response; therefore, drugs that stabilize their levels by targeting the soluble epoxide hydrolase (sEH), an enzyme responsible for their metabolism, are currently under investigation. The effect of sEH inhibitors on molecular components of fever mechanism, i.e., on synthesis of pro-inflammatory cytokines or prostaglandins, has been repeatedly proven; however, the hypothesis that sEH inhibitors affect febrile response has never been tested. The aim of this study was to examine if sEH inhibition affects core body temperature (Tb) as well as Tb changes during febrile response to infectious (lipopolysaccharide; LPS) or non-infectious (turpentine; TRP) stimuli. Male Wistar rats were implanted intra-abdominally with miniature biotelemeters to monitor Tb. A potent sEH inhibitor 12-(3-adamantan-1-yl-ureido)-dodecanoic acid (AUDA) was suspended in olive oil and administrated into animals in the intraperitoneal (i.p.) dose of 15 mg/kg, which, as we showed, has no significant influence on normal Tb. We have found that AUDA injected 3 h after LPS (50 μg/kg i.p.) significantly weakened febrile rise of Tb. Moreover, injection of sEH inhibitor 7 h after turpentine (administrated subcutaneously in a dose of 100 μL/rat) markedly reduced the peak period of aseptic fever. Obtained results provide first experimental evidence that sEH inhibitors possess anti-pyretic properties. Therefore, medicines targeting sEH enzymatic activity should be considered as a complement to the arsenal of topical medications used to treat fever especially in clinical situations when non-steroidal anti-inflammatory drugs are ineffective.

## Introduction

Fever, also known as pyrexia, is a regulated rise in body temperature (Tb) that is most frequently associated with infection, inflammation, and trauma. Experimental data strongly suggest important role of cytokines, especially interleukin (IL)-1β and IL-6 and tumor necrosis factor-α (TNF-α), as endogenous mediators of this physiological response [4, 16]. Pyrogenic cytokines are involved in stimulation of acute phase proteins, activation of hypothalamic–pituitary–adrenal (HPA) axis, and initiation of the arachidonic acid (AA) liberation from membrane phospholipids [15]. Free AA is a precursor for eicosanoids—signaling molecules that exert complex control over many bodily systems [3]. The cyclooxygenase (COX) pathway of AA metabolism, which can be inhibited by non-steroidal anti-inflammatory drugs (NSAIDs) or recently developed COXIBs (selective inhibitors of inducible form of cyclooxygenase, COX-2), produces prostaglandin E_2_ (PGE_2_)—another important downstream mediator of fever and inflammation [2]. In contrast, cytochrome P-450 monooxygenase (epoxygenase)-derived metabolites of AA, namely epoxyeicosatrienoic acids (EETs), possess anti-inflammatory and anti-pyretic properties. Kozak and coworkers proved that various isomers of EETs administered into the lateral ventricle reduce lipopolysaccharide (LPS)-induced fever in rats [17]. However, EETs have a short half-life limiting their therapeutic application, ranging from seconds to minutes [10], and are rapidly conversed to far less biologically active or inactive dihydroxyeicosatrienoic acids (DHETs) by soluble epoxide hydrolase (sEH) [6]. Thus, inhibitors of the sEH (sEHi) that stabilize and increase the EET levels are increasingly studied in rodent models of various diseases [as reviewed in 21]. Moreover, sEH inhibitors have been shown to downregulate the expression of COX-2 protein and synergize well with NSAIDs towards the reduction of inflammation [26] as well as LPS-induced plasma levels of pro-inflammatory cytokines [27]. Thus, given the evidence that inhibition of sEH activity affects molecular components of fever mechanism: cytokines, prostaglandins, and EET bioavailability, the present study aimed to determine the effect of a potent sEH inhibitor 12-(3-adamantan-1-yl-ureido)-dodecanoic acid (AUDA) on Tb changes in a course of febrile response to infectious and non-infectious stimuli in rat. Inhibition of sEH enzyme constitutes innovative approach in treating fever; thereby, drugs targeting this enzyme might complete the arsenal of available medicines, especially in clinical situations where oral anti-pyretics are only marginally effective, for example in lowering elevated Tb occurring after stroke [40].

## Materials and methods

### Experimental animals

Male Wistar Crl:WI(Han) rats aged 8–12 weeks and weighing from 250 to 300 g were purchased from the Mossakowski Medical Research Centre Polish Academy of Sciences (Warsaw, Poland) and were acclimatized for 10 days before starting the experiments. Animals were kept individually in a room at constant relative humidity (60 ± 5%) and temperature (24 ± 1 °C), with a 12:12-h light–dark photoperiod, with lights on at 7:00 h. Rodent laboratory food and drinking water were provided ad libitum.

All procedures were approved by the Local Bioethical Committee for Animal Care (permission no. 9/2015).

### Temperature measurements

To monitor the core Tb, all animals were implanted intra-abdominally with temperature-sensitive miniature biotelemeters PhysioTels model TA10TA-F40 (Data Sciences International, St. Paul, MN, USA) under sterile condition [for details, see 39]. All surgical procedures were done at least 2 weeks before the start of experiments.

### Reagents and injections

Systemic inflammation was provoked by intraperitoneal (i.p.) injection of bacterial lipopolysaccharide (LPS) while local aseptic necrosis of tissues was induced with turpentine (TRP) administrated subcutaneously (s.c.). It is well established that injections of both agents provoke characteristic, reproducible febrile rise of Tb in rats [for, e.g., see 23, 31, 39].

LPS derived from *Escherichia coli* 0111:B4 (Sigma-Aldrich, St. Louis, MO, USA) was dissolved in pyrogen-free 0.9% sodium chloride (saline) to obtain the final concentration of 50 μg/mL. LPS was injected i.p. in a dose of 50 μg/kg to provoke endotoxin fever. Intraperitoneal injection of saline (1 mL/kg) was used as a control.

Aseptic necrosis of tissues was induced with undiluted turpentine oil (Elissa, Warsaw, Poland). Turpentine was injected s.c. into the right hindlimb at a volume of 0.1 mL/rat.

sEH inhibitor 12-(3-adamantan-1-yl-ureido)-dodecanoic acid (AUDA) was synthetized according to the procedure [13]. Dose of AUDA was suspended in 500 μL of olive oil, then sonicated, and vortexed to obtain homogeneous suspension. Suspensions were made individually for each animal freshly before use and injected i.p. in a dose of 5, 15, or 30 mg/kg according to the experiment. As a control, animals received i.p. injection of olive oil in a volume of 500 μL.

All rats were restrained and not anesthetized during injections. The animals were weighed before injections to determine the precise doses of LPS and AUDA.

### Anti-TNF-α antibody injection

TNF-α antibodies (rabbit polyclonal IgG anti-rat TNF-α; Thermo Scientific, Waltham, MA USA; cat. no. PRTNFAI) were injected i.p. in a dose of 50 μg/rat in a volume of 500 μL of phosphate-buffered saline 1 h prior to the injection of AUDA. Rabbit IgG (Rockland Immunochemicals, Limerick, PA, USA; cat. no. 011-001-297) was used as a control. The dose of TNF-α antibody (50 μg/rat corresponds to the dose of 200–250 μg/kg) was selected according to the results of our previous experiments [12].

### TNF-α assay

Blood was collected from anesthetized rats (mixture of ketamine/xylazine) by cardiac puncture into the solution of ethylenediaminetetraacetic acid (EDTA, Sigma-Aldrich, St. Louis, MO, USA). Plasma was separated by a centrifugation (20 min 1000×*g*) within 30 min of collection and was kept frozen at −20 °C until assay. Blood for analyses was collected an hour after LPS injection, at the time of the greatest decrease in Tb of rats observed with biotelemetry.

The levels of TNF-α were determined by a standard sandwich ELISA kit from Invitrogen (Camarillo, CA, USA cat. no. KRC3011; the minimum detectable dose of rat TNF-α is <4 pg/mL) according to the manufacturer’s instructions. Colorimetric changes in the assay were detected using the Synergy HT Multi-Mode Microplate Reader (BioTek, Winooski, VT, USA).

### Statistics

Temperature values are reported as means ± standard error mean (SEM). Five-minute temperature recordings were pooled into 30-min averages for presentation. Mean values ± SEM of TNF-α concentrations in plasma were calculated for five plasma samples, each from different animal in the experimental group, that were assayed in duplicate. ANOVA with repeated measures followed by a Tukey multiple comparison post hoc test was used to determine the differences in time-dependent patterns of temperature among groups. ANOVA followed by Tukey pairwise comparison was used to test for statistical differences among groups at individual time points as well as TNF-α contents. Differences were considered significant at *p* < 0.05.

## Results

### Dose-dependent effect of AUDA on Tb in rats

**Fig. 1:**
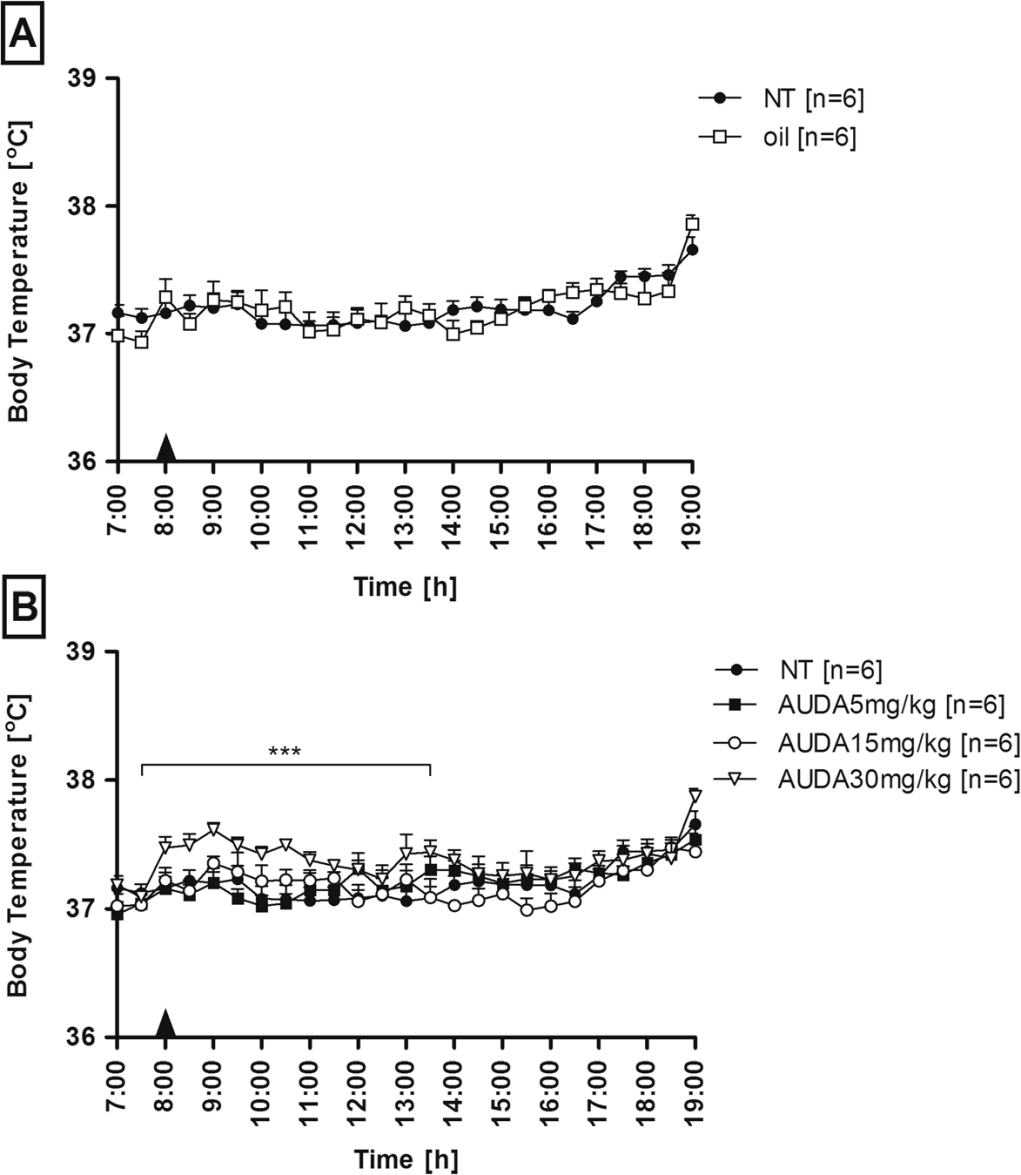
Changes of body temperature (°C) over time (h) of rats treated intraperitoneally at 8:00 with 500 μL of olive oil (*open squares*, **a**) or with AUDA (**b**) in a dose of 5 mg/kg (*closed squares*), 15 mg/kg (*open circles*), or 30 mg/kg (*open triangles*). *Closed circles* represent normal circadian rhythm of body temperature in control rats. Sample size is indicated in *parentheses*. *Black arrowheads* represent the time of injection. Values are means ± SEM at 30-min averages. *Asterisks* indicate significant difference (***p < 0.001) between rats treated with AUDA in a dose of 30 mg/kg and non-treated animals

The effect of AUDA on changes of Tb in male Wistar rats is illustrated in Fig. 1b. Three doses of sEH inhibitor (5, 15, and 30 mg/kg) were suspended in olive oil and injected i.p. into the separate groups of rats. Injection of olive oil alone (in a volume of 500 μL i.p.) did not cause significant (*p* > 0.05) changes in Tb in rats compared to non-treated animals as can be seen in Fig. 1a. Similarly, AUDA in a dose of 5 mg/kg as well as 15 mg/kg had only slender influence on Tb. Mean daytime Tb measured between 9:00 and 19:00 in control (non-treated) animals (37.21 ± 0.07 °C) was comparable (*p* > 0.05) to “olive” (37.22 ± 0.07 °C) and “AUDA 5 mg/kg” (37.24 ± 0.07 °C) as well as to “AUDA 15 mg/kg” (37.17 ± 0.06 °C) experimental groups. However, AUDA injected in a dose of 30 mg/kg visibly affected Tb of rats. Mean Tb of animals from the experimental group “AUDA 30 mg/kg” (37.43 ± 0.04 °C) was significantly (*p* < 0.001) higher between 8:00 and 13:30 compared to “NT” (37.12 ± 0.03 °C) and other groups. There were no significant (*p* > 0.05) changes in Tb between animals treated with AUDA and non-treated rats measured from 14:00 to 19:00.

Considering the obtained results, we decided to use AUDA in a dose of 15 mg/kg for further experiments as the highest dose that has not influenced the normal Tb of rats.

### AUDA weakened aseptic fever in rats

**Fig. 2:**
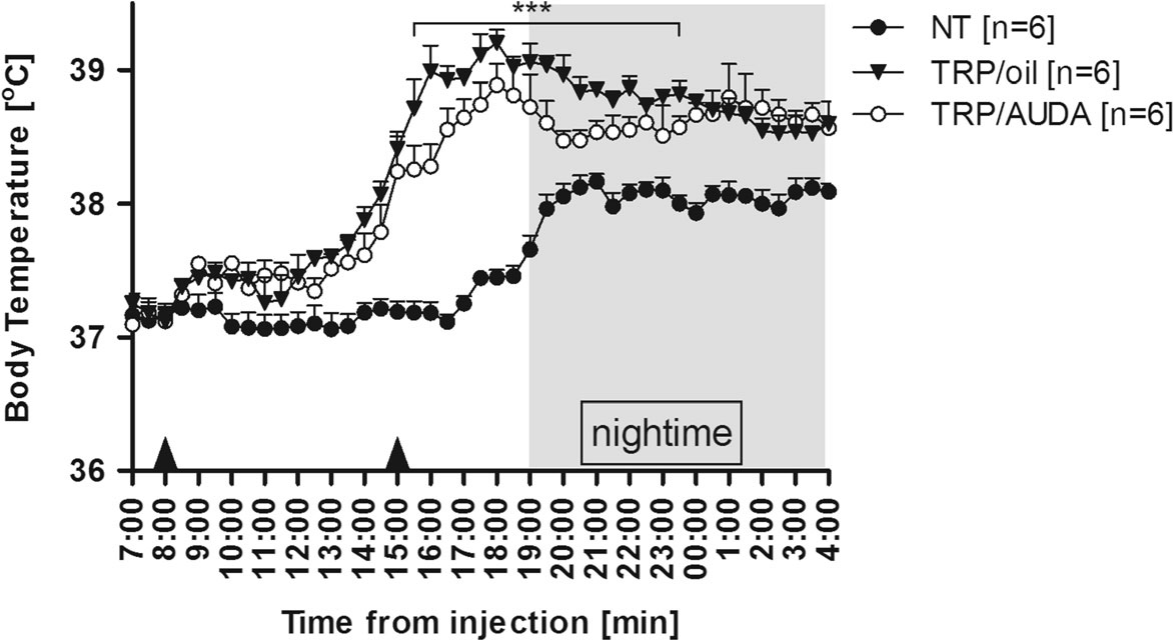
Changes of body temperature (°C) over time (h) of rats treated s.c. at 8:00 with turpentine. Seven hours afterwards, animals received intraperitoneally AUDA in a dose of 15 mg/kg (open circles) or 500 μL of olive oil (*closed triangles*) as a control. *Closed circles* represent normal circadian rhythm of body temperature in non-treated rats. Sample size is indicated in *parentheses*. *Black arrowheads* represent the time of injection. Values are means ± SEM at 30-min averages. *Asterisks* indicate significant difference (***p < 0.001) between “TRP/oil” and “TRP/AUDA” experimental groups

To determine the effect of sEH inhibition on aseptic fever, animals were injected with turpentine at time 8:00 and with AUDA or olive oil as a control after 7 h. As can be seen in Fig. 2, injection of turpentine provoke rise in Tb that started in both experimental groups with latency period lasting for c.a. 5–6 h. Then, temperature had gradually grown reaching highest values at 18:00. In comparison to rats treated intraperitoneally at 15:00 with olive oil, injection of AUDA significantly reduced the peak period of aseptic fever. Mean Tb of rats injected with oil measured between 16:00 and midnight (38.93 ± 0.05 °C) as well as maximum value of Tb rise (39.20 ± 0.09 °C at 18:00) was significantly (*p* < 0.001) higher than in animals treated with AUDA (mean Tb measured for the same time interval was 38.60 ± 0.06 °C and maximum of Tb rise reached 38.89 ± 0.15 °C at 18:00, respectively). Then, a 6-h-lasting gradual decrease of Tb towards normal was observed, which did not significantly (*p* > 0.05) differ in both experimental groups.

### AUDA weakened endotoxin fever in rats

**Fig. 3:**
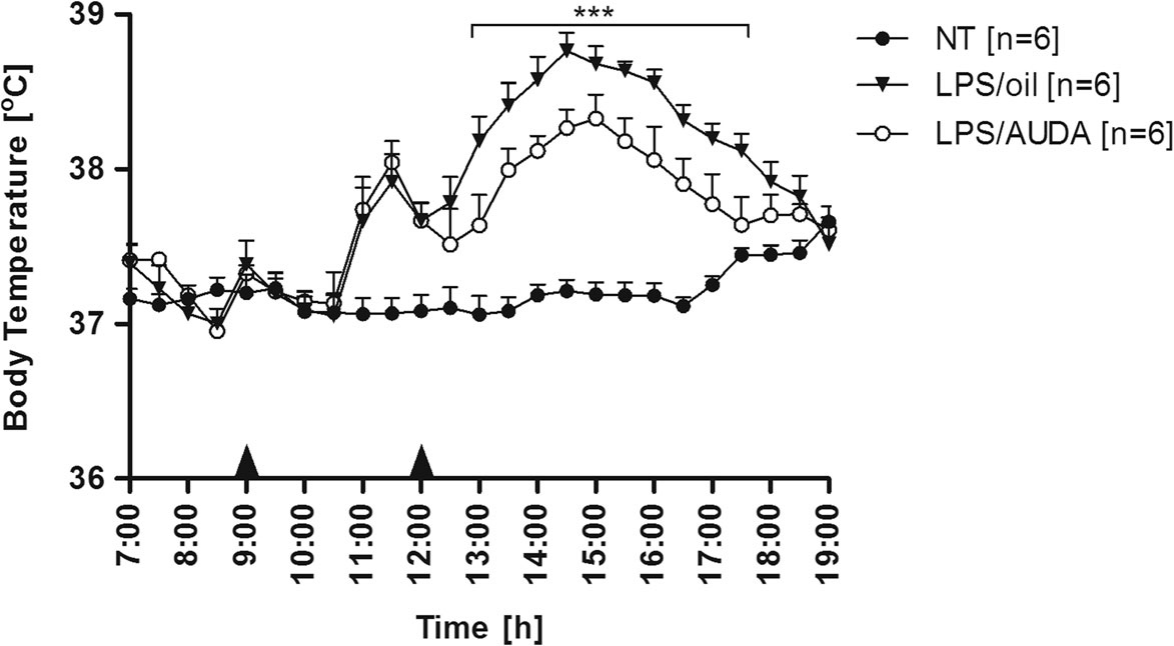
Changes of body temperature (°C) over time (h) of rats treated i.p. at 9:00 with LPS (50 μg/kg). Three hours afterwards, animals received intraperitoneally AUDA in a dose of 15 mg/kg (*open circles*) or 500 μL of olive oil (*closed triangles*) as a control. *Closed circles* represent normal circadian rhythm of body temperature in non-treated rats. Sample size is indicated in *parentheses*. *Black arrowheads* represent the time of injection. Values are means ± SEM at 30-min averages. *Asterisks* indicate significant difference (***p < 0.001) between “LPS/oil” and “LPS/AUDA” experimental groups

As can be seen in Fig. 3, i.p. injection of LPS (in a dose of 50 μg/kg) induced characteristic biphasic fever in rats that started with latency period lasting 1.5 h. The first peak of Tb rise was observed 2.5 h, while second 6 h after LPS injection in both experimental groups. Until the AUDA or olive oil injection, Tb in both groups did not differ. However, the febrile response was strongly weakened in the animals that received sEH inhibitor 3 h after LPS compared to rats injected with olive oil as a control. The maximum value of Tb rise in the “LPS/AUDA” group of rats (38.33 ± 0.15 °C) as well as mean Tb (38.03 ± 0.09 °C) measured between 13:30 and 17:30 was significantly (*p* < 0.001) lower than in the “LPS/oil” group (with Tb max 38.76 ± 0.12 °C) and mean Tb 38.47 ± 0.09 °C measured for the same time period).

### AUDA pre-injection caused drop of Tb after LPS administration

**Fig. 4:**
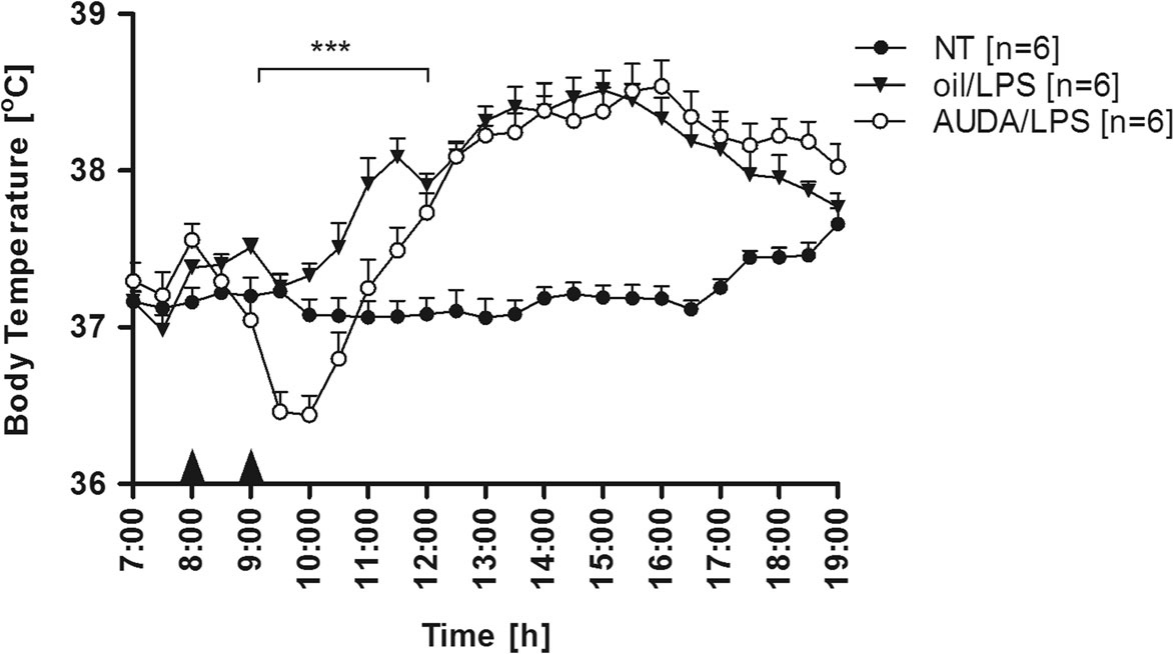
Changes of body temperature (°C) over time (h) of rats treated intraperitoneally at 8:00 with AUDA in a dose of 15 mg/kg (*open circles*) or with 500 μL of olive oil (*closed triangles*) an hour before LPS (50 μg/kg) administration. *Closed circles* represent normal circadian rhythm of body temperature in non-treated rats. Sample size is indicated in *parentheses*. *Black arrowheads* represent the time of injection. Values are means ± SEM at 30-min averages. *Asterisks* indicate significant difference (***p < 0.001) between “oil/LPS” and “AUDA/LPS” experimental groups

Figure 4 illustrates the effect of pre-injection with AUDA on endotoxin fever in rats. Pre-injection with olive oil did not change the characteristic biphasic pattern of endotoxin fever, which corresponds with a previous report from our laboratory [38]. In this experimental group, fever started an hour after LPS injection and reached first peak (38.09 ± 0.12 °C) after 2.5 h. The maximum value of Tb during the second phase of fever (38.52 ± °C) was observed 6 h post-LPS injection. Then, a 4-h-lasting gradual decrease of Tb towards normal was observed. In contrary, the animals pre-injected with AUDA responded with a drop of Tb almost instantly after LPS administration to the minimum of 36.44 ± 0.12 °C. Then, temperature rose steadily. Whereas the first phase of endotoxin fever in this experimental group was completely diminished, the second phase with a Tb maximal value of 38.54 ± 0.16 °C achieved an hour later was comparable to the “olive/LPS” group. However, for short time period (between 18:00 and 19:00), mean Tb in “AUDA/LPS” rats was significantly (*p* < 0.001) higher than in the olive/LPS group.

### AUDA did not affect the rise in plasma TNF-α after LPS injection in rats

**Fig. 5:**
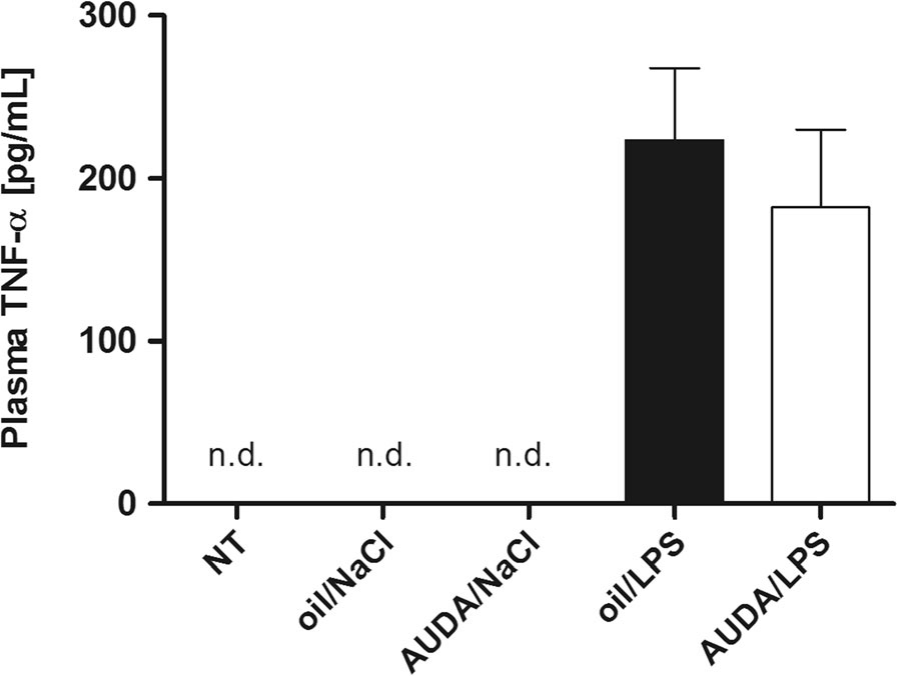
Plasma TNF-α concentration (pg/mL) measured 1 h after LPS (50 μg/kg i.p.) or saline (1 mL/kg i.p.) injection to animals pre-treated intraperitoneally with AUDA (15 mg/kg) or with 500 μL of olive oil as a control. All groups correspond to those shown in previous experiment. Values are means ± SEM calculated for plasma samples obtained from six animals for each experimental group

Plasma TNF-α concentration was measured an hour after LPS or saline injection to the rats pre-treated with AUDA or olive oil. This particular time point corresponds to the greatest decrease in Tb of AUDA/LPS rats as it was previously described. As can be seen in Fig. 5, in plasma of non-treated rats, as well as in the animals treated with saline an hour after olive oil or AUDA, TNF-α levels were below the detection range of ELISA kit used for experiments (the minimum detectable dose was 5 pg/mL). In both groups of rats treated with LPS, we found a significant increase in TNF-α levels (182 ± 48 pg/mL in plasma of animals pre-treated with AUDA and 223 ± 44 pg/mL in rats pre-treated with olive oil, respectively). However, the difference in TNF-α concentration between these two groups of animals was insignificant (*p* > 0.05).

### Injection of TNF-α antibodies did not affect the drop of Tb observed after LPS injection into animals pre-treated with AUDA

**Fig. 6:**
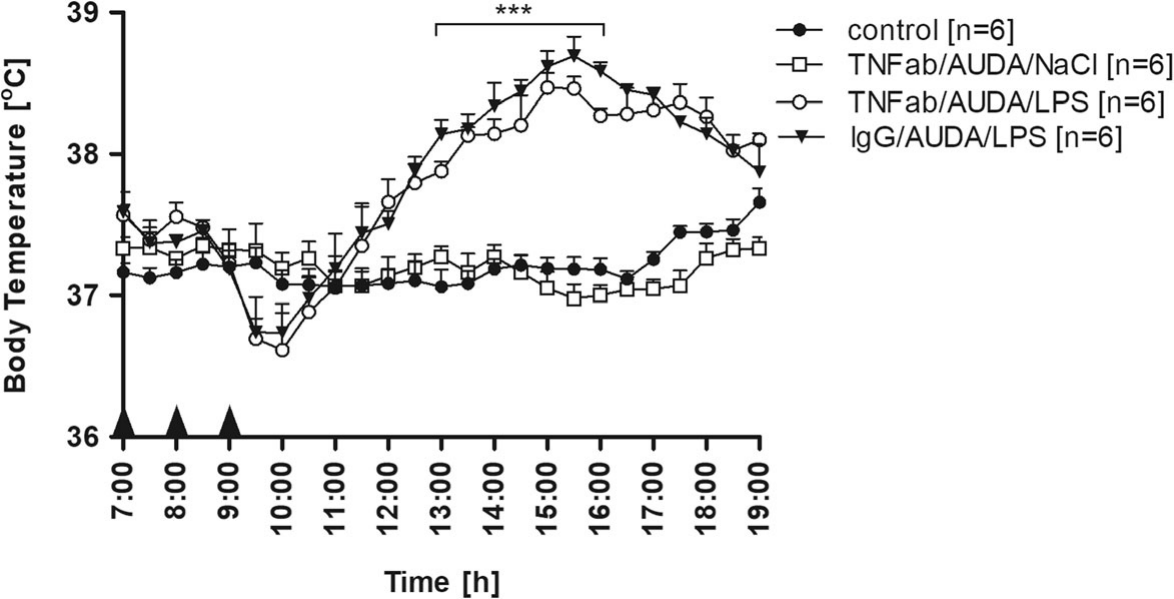
Changes of body temperature (°C) over time (h) of rats treated intraperitoneally at 7:00 with TNF-α antibodies (50 μg/rat i.p.) and at 8:00 with AUDA in a dose of 15 mg/kg an hour before LPS (50 μg/kg i.p.) (*open circles*) or NaCl (*open squares*) administration. *Closed triangles* represent Tb of rats treated at 7:00 with IgG (50 μg/rat i.p.) and at 8:00 with AUDA an hour before LPS injection (both in same concentration as above). *Closed circles* represent normal circadian rhythm of body temperature in non-treated rats. Sample size is indicated in *parentheses*. *Black arrowheads* represent the time of injection. Values are means ± SEM at 30-min averages. *Asterisks* indicate significant difference (***p < 0.001) between experimental groups “TNFab/AUDA/LPS” and “IgG/AUDA/LPS”

To determine whether or not TNF-α is involved in the drop of Tb observed after LPS injection into animals pre-treated with AUDA, in separate experiments, rats were treated i.p. with TNF-α antibodies 1 h prior to the injection of sEH inhibitor or with IgG as a control. As can be seen in Fig. 6, subsequent injections with TNF-α antibodies, AUDA, and NaCl caused any noticeable changes in Tb. Mean daytime Tb of rats from control group “TNFab/AUDA/NaCl” measured between 10:00 and 19:00 (37.15 ± 0.05 °C) was comparable (*p* > 0.05) to non-treated animals (37.21 ± 0.07 °C). It was also found that injection of TNF-α antibodies did not affect the drop of Tb observed after LPS administration to AUDA-treated animals. There were no significant (*p* > 0.05) changes in the course of febrile rise in Tb between rats from experimental groups “IgG/AUDA/LPS” and “TNFab/AUDA/LPS” within 4 h after LPS injection. In both mentioned groups of rats, characteristic pattern of endotoxin fever had changed from bi-phased to one-phased; however, in the animals treated with TNF-α antibodies, the peak period of febrile response was weakened in comparison to rats treated with IgG. The mean Tb measured between 13:00 and 16:00 in the experimental group TNFab/AUDA/LPS (38.23 ± 0.07 °C) was significantly (*p* < 0.01) lower than in the IgG/AUDA/LPS control animals (38.43 ± 0.08 °C).

## Discussion

Inhibition of sEH represents a novel therapeutic strategy to treat hypertension and inflammation and to reduce pain [28]. However, the usefulness of sEH inhibitors as anti-pyretic drugs has not been examined yet. The present study aimed to test whether sEH inhibition affects the normal Tb and Tb changes in the course of fever in rats. In the experiments, we used 12-(3-adamantan-1-yl-ureido)-dodecanoic acid (AUDA)—a urea-based potent sEH inhibitor most extensively described in the literature [for, e.g., see 5, 19, 29]. We found that AUDA injected in a dose of 5 mg/kg as well as 15 mg/kg had no significant influence on Tb of rats (as can be seen in Fig. 1b), whereas 30 mg/kg induced meager but significant rise in Tb during early hours after injection. There are no experimental data that would clearly explain such result. To date, the testing in rodents and cell culture has found the urea-based sEH inhibitors to have extremely low toxicity and no adverse effects have been observed when treating rodents chronically [9]. However, to the best of our knowledge, Tb of animals treated with sEH inhibitors has never been measured and AUDA was never injected in such high intraperitoneal dose in animal studies. Since this compound does not directly affect phospholipase A_2_, an enzyme that releases AA from membrane phospholipids [21] and without exogenous stimuli that induces liberation of AA, injection even a massive dose of sEH inhibitor should not lead to an increase in EET levels. Nevertheless, it was recently discovered that EETs constitute a substrate for COX leading to formation of corresponding epoxy-prostaglandins [24]. This should be considered especially when major pathway of EET metabolism (by sEH) is inhibited. Verification of the hypothesis that injection of high dose of sEH inhibitor leads to an increase in epoxy-prostaglandin level in the circulation and that it translates into an increase in Tb requires, however, separate studies. Therefore, in all the following experiments, we used AUDA in a dose of 15 mg/kg. As we also established, this particular dose had no significant influence on hematological parameters in rats (data not shown).

To examine whether sEH inhibition affects fever, AUDA was given an hour before injection of LPS or turpentine as well as in the course of febrile response to these compounds. Not surprisingly, AUDA injected an hour before TRP had no influence on feverish changes in Tb (data not shown). Turpentine-induced fever is characterized with long (lasting at least 5–6 h) latency period, and as we presume, sEH inhibitor was biologically inactivated before it could affect endogenous components of fever mechanism. It is known that with the adamantine sensitive to P450 oxidation and the fatty acid chain sensitive to β-oxidation, AUDA is rapidly metabolized in vivo [21]. However, when inhibitor was administrated 7 h after turpentine injection, it significantly reduced the peak period of fever in rats (Fig. 2). A similar effect was observed when AUDA was injected 3 h after LPS, in experimental model of systemic inflammation (Fig. 3). These results provide the first experimental evidence that sEH inhibitor AUDA possesses anti-pyretic properties.

**Fig. 7:**
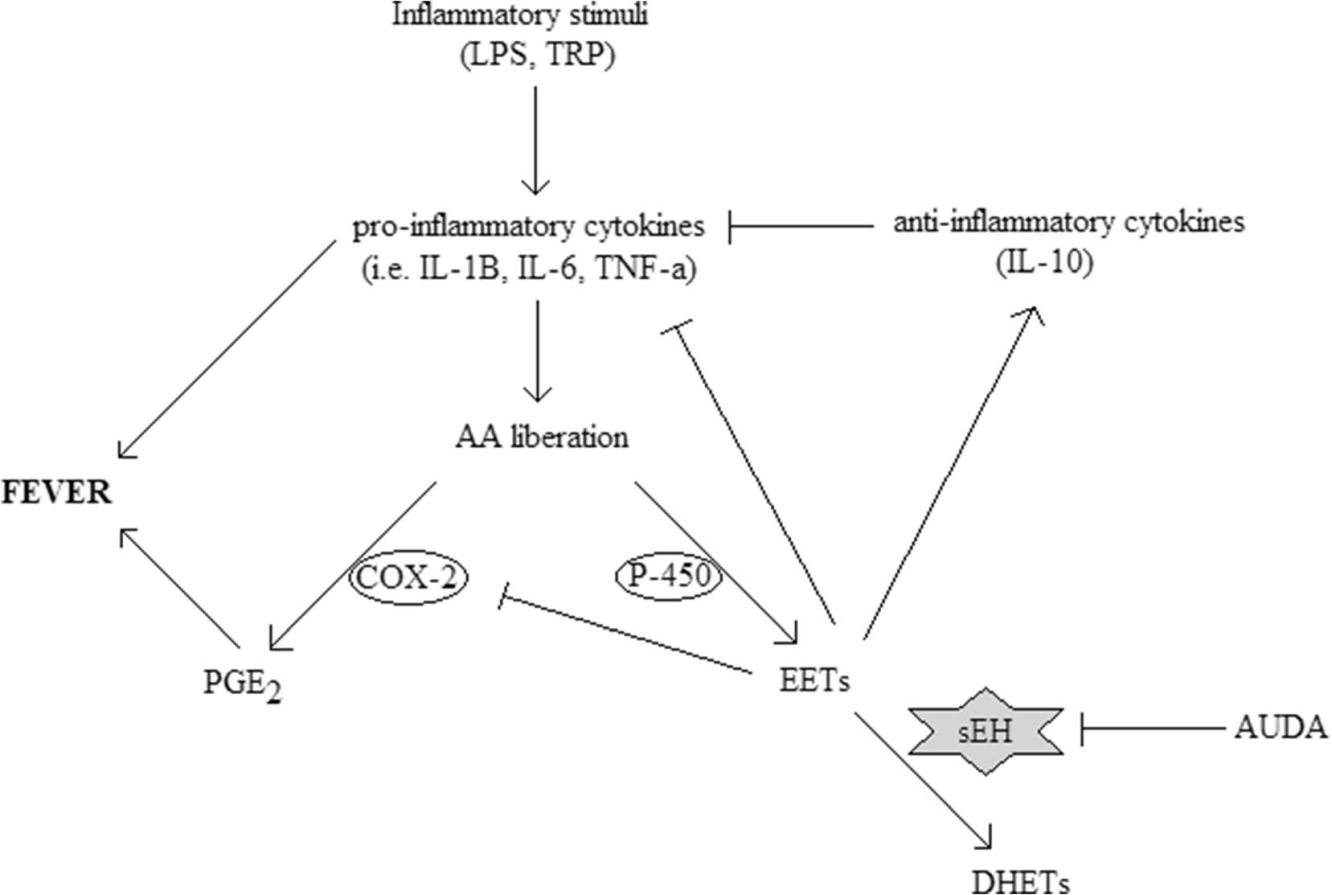
Effects of EETs increased by sEH inhibition with AUDA on the main components of molecular mechanism of fever. On the diagram, ↓ *arrowheads* represent activation while ⊥ inhibition. As a result of AUDA administration in the course of febrile response to inflammatory stimuli, DHET formation is inhibited and EETs produced from arachidonic acid by cytochrome P-450 monooxygenase are increased and available for a prolonged period. EETs acting by the mechanisms described in the discussion section lead to downregulation in fever mediators—cytokines and prostaglandins—thereby weakening fever

We believe that the reduction in fever caused by AUDA administration was the result of an increase in EET concentration that leads predominantly to pro-inflammatory cytokine downregulation and a decrease in the level of COX-2-derived PGE_2_ (as illustrated in Fig. 7). This assumption is based on the earlier studies concerning the influence of this drug on mechanisms involved in the febrile response. Tao et al. showed that the LPS-induced elevation of sEH activities was significantly reduced in AUDA-treated mice. Moreover, in comparison with vehicle (LPS-treated group), administration of AUDA (10 mg/kg i.p.) markedly increased EET level and dampened the activation of nuclear factor (NF)-κB in LPS-challenged mice [34]. These findings are consistent with the previous reports indicating that EETs prevent amplification of inflammatory signaling pathways by inhibition of transcription factor NF-κB and IκB kinase [22]. Without NF-κB translocation to the nucleus, immune cells would not produce pro-inflammatory proteins, including TNF-α, IL-6, and COX-2 [27]. Furthermore, it was reported that AUDA enhances the anti-inflammatory effects in endothelial cells by increasing the EET-induced peroxisome proliferator-activated receptor gamma (PPARɣ) activity [20]. Activation of PPARγ negatively influences the production of inflammatory cytokines, such as TNF-α, IL-6, and IL-1β, by macrophages [7]. Rodent studies strongly support our assumption. In a murine model of renal ischemia reperfusion injury, pro-inflammatory cytokines TNF-α and monocyte chemoattractant protein-1 (MCP-1) were significantly suppressed, while anti-inflammatory IL-10 and transforming growth factor-β (TGF-β) were enhanced by treatment with AUDA (10 mg/kg i.p.) as shown by real-time PCR. Furthermore, AUDA caused a decrease in plasma levels of another pro-inflammatory cytokine IL-6, while the concentrations of regulatory cytokines IL-4 and IL-10 were augmented [18]. Similarly, Schmelzer et al. showed that AUDA-butyl ester (AUDA-BE) metabolized in vivo to the AUDA decreased plasma levels of pro-inflammatory cytokines (IL-6 and TNF-α) in mice injected with LPS [27]. Further work from this laboratory demonstrates also that when administered alone, AUDA-BE decreased the protein expression of COX-2 and was more effective in decreasing PGE_2_ levels in LPS-challenged mice than NSAIDs [26].

Interestingly, we found that AUDA injected an hour before LPS caused significant and rapid drop of Tb that almost completely diminished the first phase of fever (as can be seen in Fig. 4). Initially, we assumed that observed effect results from the TNF-α upregulation. TNF-α is the first cytokine that appears after LPS administration, peaks after 1–2 h, and can exert both pyrogenic or anti-pyretic effects [1, 12, 14]. Surprisingly, we found no significant increase in plasma TNF-α concentration measured 1 h after LPS administration to animals pre-treated with AUDA compared to vehicle (Fig. 5). Furthermore, injection of TNF-α antibodies before AUDA did not protect against observed drop in Tb in LPS-challenged rats (Fig. 6), thus indicating that the discussed effect is TNF-α-independent. Therefore, we assume that a drop in Tb after LPS injection into the animals pre-treated with AUDA might result from the sudden decrease in blood pressure (BP). It is generally accepted that LPS in high intraperitoneal doses (greater than 1 mg/kg) provokes a decrease in blood pressure in rats [35], but still, little is known of the effect of low doses corresponding to those used in our experiments. Soszynski and Krajewska showed that LPS (50 μg/kg) injected i.p. into rats causes a significant increase in plasma level of potent vasodilator nitric oxide (NO) within 3 h [32]; however, some reports suggest that this effect does not have to translate into lowering blood pressure [25]. As we mentioned, recent findings showed that AUDA significantly elevates levels of vasorelaxing EETs in LPS-treated mice [34]. In regard to interactions between EETs and NO, both appear to act independent; nevertheless, it was proved that EETs activate endothelial isoform of nitric oxide synthase (eNOS) [8]. In the light of these results, we presume that the observed drop in Tb is a consequence of a sudden increase in vasorelaxing agents as a consequence of synergizing effect of AUDA and LPS.

Inhibition of sEH is an emerging strategy for treatment of cardiovascular and inflammatory disorders. We showed that sEH inhibitors should also be considered as potential anti-pyretic drugs. Undoubtedly, non-steroidal anti-inflammatory drugs (NSAIDs) are one of the most widely prescribed medications in the world for treating fever. The major problem with the use of these drugs is that their chronic administration is limited by the metabolic and cardiovascular side effects [30, 33]. Moreover, NSAIDs are only marginally effective in lowering elevated Tb in specific clinical cases, i.e., in treating fever occurring after stroke that is often associated with poor outcomes [40]. We have previously proved in the animal model of cerebral hemorrhage that such rise in Tb is a PGE2-dependent response, however, it appeared to be not sensitive to a COX inhibitor, unlike the fever induced by LPS [37]. Drugs targeting sEH not only are safe in use, but also reduce the undesirable side effects and synergize well with NSAIDs and COX-2 blockers (COXIBs) [28]. They might be attractive in drug combinations allowing to reduce the dose of anti-pyretics used in clinics. Increased circulating levels of EETs caused by sEH inhibition reduce not only NF-κB nuclear translocation but also COX-2-dependent synthesis of PGE_2_ in both peripheral tissues and central nervous systems [36]. Furthermore, recent findings showed that sEH inhibitors could eliminate pain caused by the injection of the PGE_2_ that cannot be treated with either NSAIDs or steroids [11]. It clearly shows that sEH inhibitors by stabilizing EETs affect the biology of prostaglandins through several mechanisms that are not fully understood and involvement of receptors and signaling pathways cannot be ruled out as well [41]. Further studies should concentrate on investigating the usefulness of newly discovered sEH inhibitors in combination with NSAIDs especially in clinical cases where topical therapy does not work.

## Conclusion

The results of the present study clearly demonstrate that a potent sEH inhibitor AUDA injected in a course of endotoxin or aseptic fever significantly weakened the rise in Tb of rats. This effect of AUDA undoubtedly results from the increase in EET bioavailability that leads to an inhibition of NF-κB transcriptional activity and COX-2 enzymatic activity and in consequence to downregulation of fever mediators—cytokines and prostaglandins. Obtained results constitute the first experimental evidence that sEH inhibitors should be considered as potential anti-pyretic drugs and thereby should be further examined for their suitability in clinics. Since inhibitors of sEH synergize well with NSAIDs, combined therapy could allow to reduce the side effects resulting from chronic intake of NSAIDs or to increase the effectiveness of treatment, especially of patients who experience stroke. The present study provides also a new experimental model for studying the biological effects of newly synthesized sEH inhibitors and may as well contribute to the expansion of therapeutic area of interest for sEH inhibitors.
